# The longitudinal NIHR ARC North West Coast Household Health Survey: exploring health inequalities in disadvantaged communities

**DOI:** 10.1186/s12889-020-09346-5

**Published:** 2020-08-18

**Authors:** Clarissa Giebel, Jason C. McIntyre, Ana Alfirevic, Rhiannon Corcoran, Konstantinos Daras, Jennifer Downing, Mark Gabbay, Munir Pirmohamed, Jennie Popay, Paula Wheeler, Keith Holt, Timothy Wilson, Richard Bentall, Ben Barr

**Affiliations:** 1 Department of Primary Care & Mental Health, University of Liverpool, Waterhouse Building B Block, Brownlow Street, Liverpool, L69 3GL UK; 2 NIHR ARC NWC, Liverpool, UK; 3 School of Natural Sciences and Psychology, Liverpool John Moores University, Liverpool, UK; 4 Department of Molecular and Clinical Pharmacology, Institute of Translational Medicine, University of Liverpool, Liverpool, UK; 5 Department of Geography and Planning, University of Liverpool, Liverpool, UK; 6 Department of Health Research, Lancaster University, Lancaster, UK; 7 Department of Psychology, University of Sheffield, Sheffield, UK

**Keywords:** Health inequalities, Deprivation, Mental health, Housing, Health care utilisation, Co-production

## Abstract

**Background:**

The Household Health Survey (HHS) was developed to understand the socioeconomic determinants of mental and physical health, and health inequalities in health and social care. This paper aims to provide a detailed rationale of the development and implementation of the survey and explore socio-economic variations in physical and mental health and health care.

**Methods:**

This comprehensive longitudinal public health survey was designed and piloted in a disadvantaged area of England, comprising questions on housing, physical health, mental health, lifestyle, social issues, environment, work, and finances. After piloting, the HHS was implemented across 28 neighbourhoods – 10 disadvantaged neighbourhoods for learning (NfLs), 10 disadvantaged comparator sites, and eight relatively advantaged areas, in 2015 and 2018. Participants were recruited via random sampling of households in pre-selected neighbourhoods based on their areas of deprivation.

**Results:**

7731 residents participated in Wave 1 (*N* = 4319) and 2 (*n* = 3412) of the survey, with 871 residents having participated in both. Mental health, physical health, employment, and housing quality were poorer in disadvantaged neighbourhoods than in relatively advantaged areas.

**Conclusions:**

This survey provides important insights into socio-economic variations in physical and mental health, with findings having implications for improved care provision to enable residents from any geographical or socio-economic background to access suitable care.

## Background

Areas with different levels of wealth and opportunity are typically subject to large inequalities in health outcomes [1]. A combination of social and economic circumstances, such as high unemployment rates and high levels of chronic illness and disability in poor neighbourhoods, can contribute to poor access to healthcare services, which can negatively impact health outcomes [2]. Indeed, residents of the poorest neighbourhoods in England have a shortened average life expectancy of eight years less compared to those living in the wealthiest parts of the country [3]. This life expectancy inequality between neighbourhoods is predicted to rise [4]. The costs of those health inequalities in Europe alone equate to approximately 20% of health services in middle- and high-income countries [5]. Given the human and financial costs of this inequality, there is a need to conduct robust research to inform interventions and public health policy. Here, we describe details of a large public health survey (Household Health Survey; HHS) designed to explore and explain health inequalities, noting the key outcomes of the research.

Socio-economic status (SES) is one of the primary predictors of health inequalities [2, 6–8], and is closely linked to poorer mental and physical health [9, 10]. A recent survey offered insights into health and lifestyle factors associated with deprivation [11]. However, in order to understand the social and economic determinants relating to physical and mental health issues, there is a clear need to conduct a comprehensive survey with input from varied stakeholders and disciplines. As stated in the Marmot review [5], an active reduction in health inequalities requires addressing all social determinants of health, including education, occupation, income, the home environment and the community. With the development of measures such as The Health Inequalities Assessment Tool (HIAT) [12] and the research infrastructure we’ve developed for co-production and community involvement; comes the opportunity to assess the health inequality implications of proposed research by undertaking relevant comprehensive data collections from households in areas participating in the programme and matched areas. Considering the high levels of socio-economic disadvantage in the North West Coast (NWC) region of England [13], this geographical area was highly suitable for such a survey.

Trends in life expectancy in both men and women have increased steadily in the UK as a whole for over 150 years. However, these improvements are stalling at best in most areas in the last decade, and falling in some of the most deprived areas. Differences in average life expectancy is almost 10 years among men and nearly 8 years among women between the most and least deprived neighbourhoods (Marshall et al., 2019). Statistics demonstrate a long-term North South divide in life expectancy and inequity trends (Barr et al., 2017; ONS, 2019). The North West of England historically and recently vies with the North East for the lowest average absolute and healthy life expectancies and gaps between the highest and lowest areas within the regions. Within the area covered by this survey healthy life expectancy can vary by up to 20 years between the least and most deprived neighbourhoods, with Blackpool having the lowest healthy life expectancy of any local authority area in England. These gaps are increasing and are considered to result from systemic socio-economic differences between populations (Marmot et al., 2020).

The HHS combines data on physical and mental health, social factors, environmental factors, self-reported medications, as well as geographical information, thereby exploring the variety of determinants of health inequalities [1, 14]. The overall HHS aimed to investigate a range of objectives all pertinent to reducing health inequalities, including:

- To understand the geographic and socioeconomic determinants of mental and physical health in mostly disadvantaged neighbourhoods.
- To understand health inequalities in the utilisation of health and social care.
- To inform the integration and design of better health and social care services.
- To provide a baseline for policy and person-level implementation projects within neighbourhoods.
- To provide a vehicle for capacity building and knowledge exchange.

The aim of the present study was to explore the overarching mental and physical health, social support, housing, and other public health factors of participants from disadvantaged and less disadvantaged neighbourhoods captured in the longitudinal HHS.

## Methods

### Survey development

**Fig. 1:**
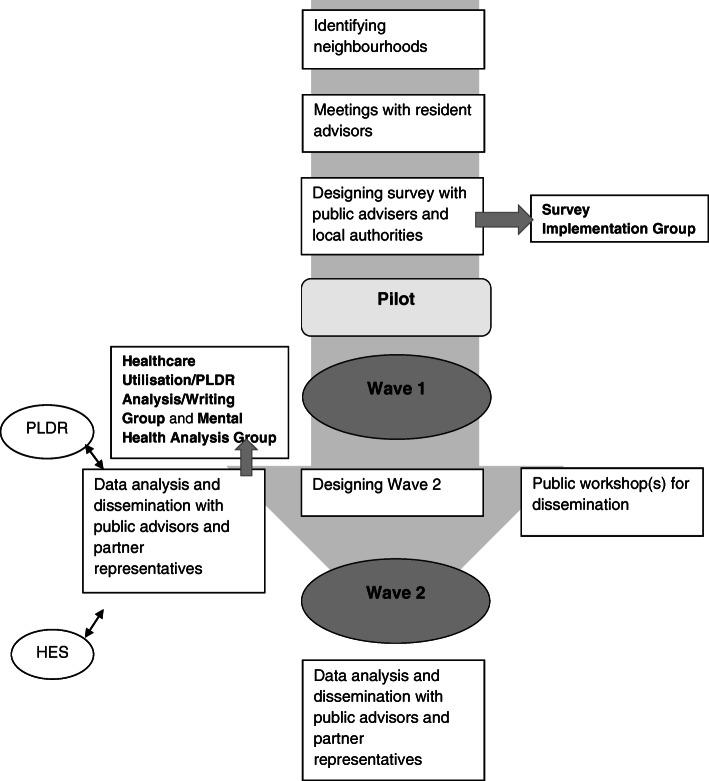
Flowchart of different stages of Household Health Survey. HES=Hospital Episode Statistics; PLDR = Place-based Longitudinal Data Resource

The NWC HHS is coordinated by researchers at the University of Liverpool and funded by the NIHR Collaboration for Leadership in Applied Health Research and Care (CLAHRC) North West Coast (NWC). The survey development was an iterative and collaborative process involving the core Applied Research Collaboration (ARC) NWC HHS team, local authorities, NHS clinicians, members of the public, acting in the capacity of public advisers and a private research company (BMG Research). Public advisers were involved in the development of the survey (TW, KH). Figure 1 shows a flowchart, which illustrates the steps involved from the design stage through to the pilot and data collection, analysis, and dissemination.

### Survey process

BMG Research conducted all data collection. Ethical approval was obtained from the University of Liverpool (Ref: RETH000836). Sampled households were mailed a letter and information leaflet at least two weeks before being approached for an interview. Interviewers then approached residents and potential participants by knocking on the resident’s door up to five times on different days and at different times of the day until it was considered as a non-response. All interviewers were trained in conducting the interview via a one-day training course. Survey interviews for the first wave were conducted on a face-to-face basis at the respondent’s homes between mid-August 2015 and early January 2016, and for the second wave between August 2018 and December 2018. All addresses were loaded electronically on to Computer Aided Personal Interviewing (CAPI) units, so that all contact information could be effectively monitored, whilst also reducing the scope for interviewer error with ID numbers, names and addresses already pre-loaded. Interviews lasted on average around 45 min.

Prior to the full survey, a pilot survey was conducted to establish any necessary changes to the methodology. For the pilot, 36 residents from NfLs and two residents from relatively advantaged areas participated. Findings from the pilot led to minor amendments of survey documents.

### Participants and recruitment

**Fig. 2:**
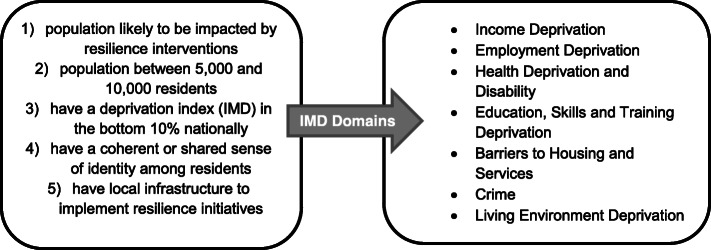
Deprivation criteria applied in the sample selection. To be considered a disadvantaged area, the neighbourhood had to meet the above five criteria. These referred to the Index of Multiple Deprivation (IMD) from the English Indices of Deprivation (ONS, 2015)

Participants were recruited from 28 neighbourhoods across the North West Coast of England in Wave 1 (2015) via random sampling of individual households. This is a region with some of the most disadvantaged neighbourhoods in the country as well as some of the most advantaged neighbourhoods, and is therefore subject to some of the greatest health and care inequalities [13]. Twenty disadvantaged neighbourhoods were identified by local authority (LA) partners. Ten of these (across eight LA areas) were subsequently identified as CLAHRC NWC’s Neighbourhoods for Learning (NfLs) where programmes of action research focused on improving the resilience of the wider health determinants governance system (known as the System Resilience Programme) were to be developed and implemented. This involved a partnership between academics, LAs and residents. The aim was to utilise research evidence alongside the experiential knowledge of those who live and work in these neighbourhoods to enhance resilience and thus address social, economic, and environmental determinants of health inequalities. Data from the HHS has been utilised in the early phases of the System Resilience Programme to support local stakeholders in identifying local issues for action, and it is being evaluated using a mixed-method approach including longitudinal data from Waves 1 and 2 of the HHS. Figure 2 shows the criteria necessary to be considered as a disadvantaged area for the purpose of the survey.

For Wave 2 (2018), only the 20 disadvantaged neighbourhoods were surveyed. Firstly, participants from Wave 1 who had expressed an interest in taking part in Wave 2 were contacted to participate. Where participants were no longer interested in a follow-up survey, or had moved away, researchers contacted other participants from the same household or residence for participation. Alternatively, new residents in the neighbourhoods were approached for participation.

### Survey data

Table 1 shows the list of questions and scales within the HHS, which included sections on demographics, housing, physical health, mental health, lifestyle, social issues, neighbourhood environment, health and social care use, and work and finances. Specific health and mental health measures include the Personalised Health Questionnaire 9 (PHQ9) [15], the Generalised Anxiety Disorder Assessment 7 (GAD7) [16], the Warwick-Edinburgh Mental Wellbeing Scale (WEMWBS) [17], and the EQ-5D [18]. The survey was newly designed and is attached in Supplementary 1.

**Table 1:** Measures included in the Household Health Survey

CATEGORY	MEASURES
Demographics	Age, Gender, Relationship status, Education, Employment, Ethnicity, Religion, Sexuality, Postcode
Physical health	Medication, Chronic illness, Multimorbidity, Self-care ability, Ability to perform usual activities, Pain, Hospital and GP visits, other health care utilisation, Physical measures
Lifestyle	Smoking, alcohol consumption, Exercise, Physical effort at work
Mental health	Well-being (sWEMWEBS), Depression, Anxiety (GAD-7), Auditory hallucinations (LSHS4), Empathy (EQ), Paranoia, Dark Triad, Rumination, Hope/Hopelessness, Locus of control, Threat anticipation, Self-esteem
Psychological	Self-control, Social Capital, Altruism, Supporting friends and family
Housing	Housing status, Condensation, Heating, Maintenance
Social issues	Observed drunkenness, observed rubbish, observed vandalism, observed racially motivated attacks, observed teenage loitering, observed troublesome neighbours, collective action
Environment	Use of public areas, Internet usage
Work and finance	Position, size of workplace, salary, hours of work, financial struggle, debt problems

Legend. EQ – Empathy Quotient scale; GAD-7 – General Anxiety Disorder scale; LSHS4 – Launay-Slade Hallucination Scale; sWEMWEBS – Warwick-Edinburgh Mental Wellbeing Scale;

### Data analysis

Data were prepared using weighting adjustment in applying survey weights to account for the differential sample sizes in each area, and to account for over- and under-response rates in certain areas which are not representative of that area’s population. Weights were applied by ward/ Lower Level Super Output Area (LSOA) using the following auxiliary variables in the following order: gender, ethnicity, economic status, and age; followed by a rim weight by population within each ward/LSOA.

**Fig. 3:**
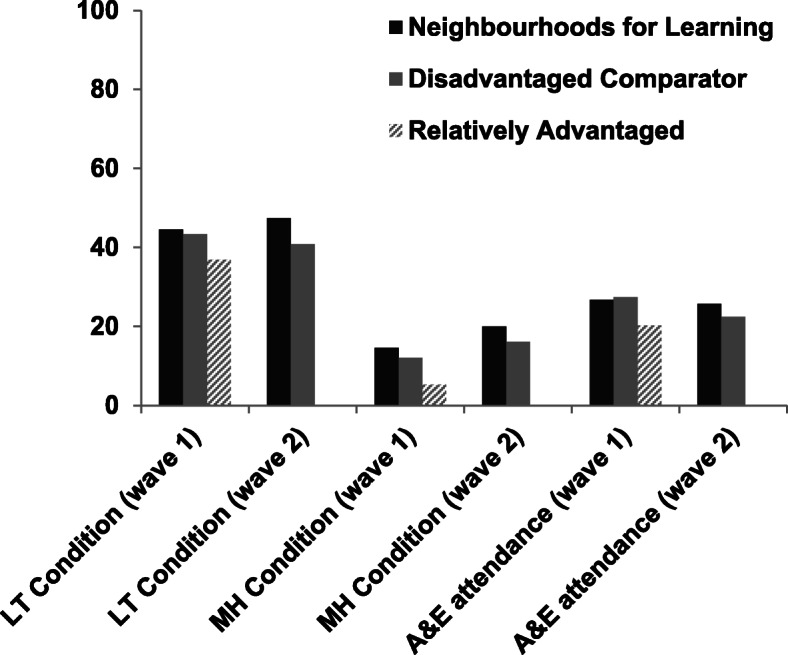
Percentage of people with Long-term conditions, mental health conditions, and who have attended A&E in the past 12 months across neighbourhood types

To test the quality of the sampling we conducted a series of parametric (ANOVA) and non-parametric (Chi-square test of independence) tests to determine whether there were differences between the NfLs, disadvantaged comparator neighbourhoods and relatively advantaged neighbourhooods, as well as between waves, on key demographic, socioeconomic, and health-related variables. Neighbourhood differences on key health and service use measures are shown in Fig. 3. ANOVAs were employed for continuous variables to detect mean differences between neighbourhood types. Follow-up comparisons employed Bonferroni adjustment to control for familywise error associated with conducting multiple tests. Chi square tests for independence were used to compare frequency statistics. This analysis tests whether the observed frequencies differ from expected frequencies if there was no relationship between neighbourhood type and the variable of interest. In other words, it tests whether there are more or less people in a particular type of neighbourhood (NfL, disadvantaged comparator, less disadvantaged) with a particular characteristic (e.g., a mental health condition) than would be *expected due to chance alone*. The significance of chi-square follow-up tests was assessed using chi-square tables, hence exact *p*-values are not reported. We also compared results to national statistics where appropriate and if data were available. Data were analysed in STATA version 14.

## Results

In total, 7731 visits were conducted in Wave 1 and 2 of the survey. Specifically, 4319 residents participated in Wave 1 of the survey (NfLs = 2009; disadvantaged comparator neighbourhoods =1501; relatively advantaged neighbourhoods = 809), which is an overall adjusted response rate of 61% of the households approached by the survey team. Of those residents that answered the door, 63.6% responded to the survey, and 32% refused in the NfLs; 57.9% responded and 36.4% refused to participate in the comparator sites; and 58.1% responded and 35.7% refused to participate in the relatively advantaged areas. Of the 10 initially identified NfLs, two failed to implement System Resilience Programme interventions and one has since dropped out from the survey. However, these neighbourhoods were maintained as NFLs in the analysis. 3412 residents participated in Wave 2, of which 871 (20.2%) residents had been followed up from Wave 1. This included 2026 (59.4%) participants from the NfLs and 1386 (40.6%) from the disadvantaged comparator neighbourhoods. With unequal variances assumed, participants who completed both waves of the survey (*M* = 51.73, *SD* = 17.40) were significantly older than participants who only completed the wave 1 survey (*M* = 48.42, *SD* = 19.51; *t* (1405.93) = 4.70, *p* < .001. Chi-square tests indicated participants completing both waves were also more likely to identify as female χ^2^ (1, *N* = 4319) = 15.43, *p* < .001, were less likely to be in paid employment (χ^2^ (1, *N* = 4319) = 26.34, *p* < .001) and were more likely to have a long-term health condition, χ^2^ (1, *N* = 4319) = 48.56, *p* < .001.

No participants were missing more than 50% of data and the level of missing data for each participant ranged from 3 to 18%. The average amount of missing data per participant was 10.75%. Of the 376 total variables, 300 variables had complete data and 9.88% of all data points were missing. Data were not missing completely at random (Little’s MCAR χ^2 =^ 7773.03, p < .001). Listwise deletion was used to account for missing values.

### Demographics

Demographic characteristics for each neighbourhood type are reported in Table 2. In Wave 1, the majority of the total sample were female (57.1%), between 25 and 34 years old (17.9%), and from a white ethnic background (89.4%). Gender [χ^2^(2, *N* = 4319) = 4.44, *p* = 0.109] and ethnicity [χ^2^(2, *N* = 4319) = .684, *p* = 0.710] did not differ by neighbourhood type. However, age was not consistent across neighbourhood type, *F* (2,3857) = 25.47, *p* < .001. Follow-up comparisons with Bonferroni adjustment indicated that participants in relatively advantaged neighbourhoods (*M* = 53.48, *SD* = 18.30) were significantly older than participants in the NfLs (*M* = 47.51, *SD* = 19.43; *t* = 5.97, *p* < 0.001) and disadvantaged comparator (*M* = 49.00, *SD* = 18.79; *t* = 4.48, *p* < .001) neighbourhoods.

**Table 2:** Descriptive characteristics by area type for Waves 1 and 2

	Neighbourhoods for Learning Wave 1 (***n*** = 2009)	Neighbourhoods for Learning Wave 2 (***n*** = 2026)	Disadvantaged Comparator areas Wave 1 (***n*** = 1501)	Comparator areas Wave 2 (***n*** = 1386)	Relatively Advantaged areas Wave 1 (***n*** = 809)
Age, N(%)					
18–24 yrs	240 (12.0)	210 (10.4)	128 (8.53)	116 (8.4)	53 (6.55)
25–34 yrs	391 (19.4)	370 (18.3)	284 (18.92)	266 (19.2)	98 (12.11)
35–44 yrs	325 (16.2)	308 (15.2)	226 (15.06)	229 (16.5)	114 (14.09)
45–54 yrs	302 (15.0)	316 (15.6)	245 (16.32)	211 (15.2)	143 (17.68)
55–64 yrs	262 (13.0)	302 (14.9)	248 (16.52)	204 (14.7)	129 (15.95)
65–74 yrs	271 (13.5)	297 (14.7)	186 (12.39)	206 (14.9)	159 (19.65)
75+	218 (10.9)	218 (10.8)	183 (12.19)	150 (10.8)	112 (13.84)
Gender, N(%)					
Male	856 (42.6)	910 (44.9)	625 (41.6)	580 (41.9)	373 (46.1)
Female	1153 (57.4)	1116 (55.1)	876 (58.4)	806 (58.15)	436 (53.9)
Ethnicity, N(%)					
White	1790 (89.2)	1800 (89.6)	1348 (90.1)	1228 (89.5)	717 (89.0)
BME	217 (10.8)	209 (10.4)	149 (9.9)	144 (10.5)	89 (11.0)
Education, N(%)					
No qualifications	903 (45.0)	844 (41.7)	613 (41.0)	542 (39.2)	202 (25.0)
Certificate	867 (43.3)	923 (45.6)	712 (47.6)	652 (47.1)	352 (43.6)
Degree/higher	235 (11.7)	257 (12.7)	170 (11.4)	190 (13.7)	254 (31.4)
Employment N(%)					
Paid	742 (37.0)	781 (38.6)	628 (41.9)	605 (44.1)	387 (48.0)
Studying	91 (4.5)	87 (4.3)	46 (3.1)	43 (3.1)	33 (4.1)
Looking for work	128 (6.4)	89 (4.4)	69 (4.6)	44 (3.2)	13 (1.6)
Unable due to illness	240 (11.9)	294 (14.5)	150 (10.0)	154 (11.2)	13 (1.6)
Retired	528 (26.3)	538 (26.6)	416 (27.7)	351 (25.6)	299 (37.1)
Homemaker	262 (13.1)	194 (9.6)	187 (12.5)	151 (11.0)	60 (7.4)
Other	17 (0.8)	40 (2.0)	4 (0.2)	25 (1.8)	2 (0.2)
Social rent N(%)	752 (37.4)	1149 (56.7)	454 (30.3)	976 (70.4)	25 (3.09)
Financial struggle N(%)					
Better off than 12 months ago	224 (11.3)	217 (10.8)	195 (13.1)	161 (11.7)	91 (11.4)
Same as 12 months ago	1403 (70.5)	1485 (73.8)	1078 (72.5)	1025 (74.6)	609 (76.0)
Worse off than 12 months ago	363 (18.2)	309 (15.4)	215 (14.4)	189 (13.8)	101 (12.6)
Caring responsibilities N(%)					
No	1736 (86.4)	1751 (86.4)	1283 (85.5)	1222 (88.2)	697 (86.1)
Yes (1–19 h/week)	111 (5.5)	110 (5.4)	102 (6.8)	71 (5.1)	62 (7.7)
Yes (20–49 h/week)	45 (2.3)	57 (2.8)	29 (1.9)	33 (2.4)	26 (3.2)
Yes (50+ h/week)	117 (5.8)	108 (5.3)	87 (5.8)	59 (4.3)	24 (3.0)

In Wave 1 there was also a significant association between education and neighbourhood type [χ^2^ (4, *N* = 4319) =232.13, *p* < .001]. The number of participants who held a degree was higher in relatively advantaged neighbourhoods [χ^2^(4, *N* = 4319) = 169.4, *p* < .01] and lower in the NfLs [χ^2^ (4, *N* = 4319) = 16.8, *p* < .01] and disadvantaged comparator neighbourhoods [χ^2^ (4, *N* = 4319) =15.1, *p* < .05]. Proportions of people in employment also varied across neighbourhood types, [χ^2^ (2, *N* = 4319) = 30.13, *p* < .001]. Examining employment as a dichotomous variable (employed, not employed), participants in relatively advantaged neighbourhoods had higher levels of employment [χ^2^ (2, *N* = 4319) =10.4, *p* < .01], while participants in NfLs had lower levels of employment [χ^2^ (2, *N* = 4319) =7.0, *p* < .05]. Observed frequencies in the disadvantaged comparator neighbourhoods did not differ from expected values [χ^2^(2, *N* = 4319) = .05, *p* > .05]*.*

Consistent with Wave 1, the majority of the sample in Wave 2 were female (56.3%), between 25 and 34 years of age (18.7%), and from white ethnic backgrounds (89.6%). Age (*t* (3123) = .10, *p* = .923), gender [χ^2^(1, *N* = 3412) = 3.15, *p* = .076], and ethnicity [χ^2^(1, *N* = 3381) = .01, *p* = .931] proportions did not differ between NfLs and deprived comparator neighbourhoods. Education level did not vary according to neighbourhood type [χ^2^(2, *N* = 3408) = 2.37, *p* = 0.306]. Employment was related to neighbourhood type in the overall chi-square test [χ^2^(1, *N* = 3396) = 10.09, *p* = .001, but no individual proportions varied significantly from expected values (all χ^2^’s < 3.7, all p’s > .05).

### Neighbourhood deprivation

Differences in deprivation at the neighbourhood level as measured by the Index of Multiple Deprivation (IMD) were examined via a one-way ANOVA (Wave 1) and an independent-samples t-test for unequal variances (Wave 2). In Wave 1, deprivation varied according to neighbourhood type, *F* (2, 4316) = 1842.92, *p* < .001. Post-hoc Least Significant Difference tests indicated that the relatively advantaged neighbourhoods (*M* = 11.32, *SD* = 7.58) had significantly lower levels of deprivation compared to the NfLs (*M* = 50.51, *SD* = 17.76, *p* < .001) and disadvantaged comparator neighbourhoods, *M* = 42.76, SD = 14.56, *p* < .001. The NfLs were also significantly more deprived than the disadvantaged comparator neighbourhoods. In Wave 2, the NfLs (*M* = 54.22, *SD* = 16.56) were significantly more deprived than the deprived comparator neighbourhoods (*M* = 42.27, *SD* = 14.54), *t* (3206.87) = 22.27, *p* < .001.

### Caring responsibilities

In Wave 1, the number of people who reported caring responsibilities for a family member, friend, neighbour or other because of long-term physical or mental ill-health or disability or problems related to old age was consistent across neighbourhood types, [χ^2^(2, *N* = 4319) = .64, *p* > .05], with the majority of respondents reporting no caring responsibilities (~ 85%). In Wave 2, the proportions of people reporting caring responsibilities did not significantly differ between NfL and deprived comparator neighbourhoods, χ^2^(1, *N* = 3412) = 3.86, *p* = .145.

### Physical health

There was a significant association between the number of people reporting long-term health conditions and neighbourhood type [χ^2^ (2, *N* = 4319) =14.08, *p* < .001]. Fewer people in relatively advantaged neighbourhoods reported having a long-term condition [χ^2^ (2, *N* = 4319) =6.3, *p* < .05]. Multimorbidity, however, did not differ between the three neighbourhood types [χ^2^ (4, *N* = 4319) =2.98, *p* = .562].

There was an association between long-term physical health conditions and neighbourhood type in wave 2 of the survey, χ^2^(1, *N* = 3389) = 14.41, *p* < 0.001. The deprived comparator neighbourhoods had significantly fewer people with long-term conditions than expected, χ^2^ (1, *N* = 3389) = 4.7, *p* < .05. A Chi-square test indicated that multimorbidity in the wave 2 sample varied by neighbourhood type, χ^2^(1, *N* = 3412) =4.94, *p* = .026. However, the follow-up analyses indicted no significant effect of neighbourhood type.

### Self-reported medicine intake by class

The percentage of people reporting use of prescription medication ranged between 1.4 and 16.8% in the whole Wave 1 sample. Analgesics (16.8%) and anti-hypertension medication (16.7%) were the most frequently prescribed classes of drugs. Anti-depressants (11.1%), lipid-lowering medication (10.5%), and asthma medication (9.7%) were each prescribed to approximately one tenth of the total sample. Proton-pump inhibitors (7.4%), anti-diabetics (5.2%), anti-platelets (5.1%), anti-bacterial (3.5%), and anti-psychotics (1.4%) were prescribed less often. Chi square tests revealed significant associations with neighbourhood type for analgesics [χ^2^ (2, *N* = 4319) = 26.20, *p* < .001] and antidepressants [χ^2^ (2, *N* = 4319) =29.28, *p* < .001]. Reports of analgesic use were lower than expected in less disadvantaged neighbourhoods, χ^2^ (2, *N* = 4319) = 16.8, *p* < 0.05. Antidepressant use was also lower than expected in the less deprived neighbourhoods, χ^2^ (2, *N* = 4319) =17.6, *p* < .05. All other medication class usage was consistent across neighbourhood types.

In Wave 2, prescription rates ranged from 1.1% (anti-psychotics) to 14.4% (anti-depressants). Anti-depressants and analgesics (13.7%) were the most frequently prescribed medication. In order of frequency, people also reported taking hypertensive (11.4%), lipid lowering (9.3%), asthma (9.3%), cardiovascular (8.9%), anti-diabetic (5.4%) proton-pump inhibitor (5.0%), anti-bacterial (2.5%), and anti-platelet medications. The rates of prescription of all medication classes did not significantly differ between NfL and deprived comparator neighbourhoods with alpha set at .05.

### Self-reported symptoms of mental ill-health

The numbers and proportions of people who reported symptoms of anxiety and depression to a level consistent with diagnosis of anxiety or depression are described in Table 2. In Wave 1, there was a significant relationship between the number of people reporting anxiety or depression and neighbourhood type [χ^2^ (2, *N* = 4317) =93.84, *p <* 0.001]. Specifically, there were significantly more than expected reports of mental health problems in the NfLs [χ^2^ (2, *N* = 4317) = 25.4, *p <* 0.01], and significantly fewer than expected reports of mental health problems in the relatively advantaged neighbourhoods [χ^2^ (2, *N* = 4317) = 46.9, *p <* 0.01]. The number of people reporting anxiety or depression in the disadvantaged comparator neighbourhoods did not differ from expected values [χ^2^ (2, *N* = 4317) =0.6, *ns*]*.*

The proportion of people reporting anxiety or depression in Wave 2 varied according to neighbourhood type, χ^2^(1, *N* = 3404) = 12.30, *p* < 0.001. Specifically, the number of people in the deprived comparator neighbourhoods reporting anxiety or depression was higher than expected, χ^2^ (1, *N* = 3404) =5.4, *p* < .05.

### Housing and environment

There was an uneven distribution of people in social housing across neighbourhood type [χ^2^ (2, *N* = 4319) = 337.21, *p* < 0.001]. Significantly more people than expected in the NfLs resided in social housing [χ^2^ (2, *N* = 4319) = 56.2, *p* < 0.01], while significantly fewer people than expected from the relatively advantaged neighbourhoods reported living in social housing [χ^2^ (2, *N* = 4319) =183.3, *p* < 0.01]. The number of people living in social housing for the disadvantaged comparator neighbourhoods did not differ from expected values [χ^2^ (2, *N* = 4319) =1.6, *ns*].

There was an overall association between “having problems with condensation” and neighbourhood type [χ^2^(2, *N* = 4205) =6.25, *p* < .05]. However, follow-up tests revealed no individual proportions were significant at the 0.05 level for any neighbourhood type. Reports of mould showed an association with neighbourhood type [χ^2^ (2, *N* = 4224) =20.77, *p* < 0.001]. The number of people reporting mould in the relative advantaged neighbourhoods was lower than expected [χ^2^ (2, *N* = 4205) =11.3, *p* < 0.01]. A similar pattern was observed when assessing the frequencies of people reporting problems with keeping warm in winter across neighbourhood types. The overall association was significant [χ^2^ (2, *N* = 4233) = 53.88, *p* < 0.001] and follow-up tests revealed that in the NfLs there were more reports of heating problems than expected [χ^2^ (2, *N* = 4205) =17, *p* < 0.01], whilst there were fewer reports than expected in the relatively advantaged neighbourhoods, χ^2^ (2, *N* = 4205) = 32.7, *p* < 0.01. The number of people who reported problems keeping warm in the disadvantaged comparator neighbourhoods did not differ from expected values [χ^2^ (2, *N* = 4205) = .3, *ns*].

Consistent with the Wave 1 data, there was an uneven distribution of people in social housing across the two neighbourhood types in Wave 2, χ^2^(1, *N* = 3412) =65.81, *p* < .001. There were significantly more people than expected living in social housing in the NfLs [χ^2^ (1, *N* = 3412) = 16.6, *p* < 0.05] and significantly fewer people than expected living in social housing in the deprived comparator neighbourhoods, χ^2^ (1, *N* = 3412) = 65.81, *p* < .05.

### Work and finances

An overall chi-square test revealed that financial struggle was associated with neighbourhood type [χ^2^ (2, *N* = 4279) =19.44, *p* = .001]. However, follow-up tests revealed that no individual proportions were significant at the 0.05 level. In Wave 2, the proportion of people experiencing various levels of financial struggles was consistent across the neighbourhood types, χ^2^(2, *N* = 3386) =3.29, *p* = .345.

### Healthcare service usage

In Wave 1, the overall percentage of people in our survey, who reported attending A&E or visiting a GP in the past 12 months was 25.75, and 69.23% respectively. 21.4% of participants visited both A&E and their GP, 51.9% visited one of these two services, and 26.3% visited neither A&E nor their GP in the previous 12 months. There was a significant association between neighbourhood type and A&E attendance [χ^2^ (2, *N* = 4307) =15.64, *p* < .001]. Follow-up tests indicated that there were lower than expected numbers of people attending A&E in relatively advantaged neighbourhoods [χ^2^(2, *N* = 4307) =9.3, *p* < .01], but proportions of A&E attendance in the NfLs and disadvantaged comparator neighbourhoods were not different from expected values. GP attendance rates were not significantly related to neighbourhood type [χ^2^ (2, *N* = 4307) =1.62, *p* = .444].

In Wave 2, 24.33% of respondents reported attending A&E in the previous 12 months and 62.43% of respondents visited their GP. A&E attendances varied according to neighbourhood type, χ^2^(1, *N* = 3402) = 4.88, *p* = 0.027, as did GP attendances, χ^2^(1, *N* = 3401) =5.80, *p* = .016. However, no individual frequency was significantly different to the expected value for either type of service use, χ^2^ ‘s < 2.3, p’s > .05. Previous analysis of the data showed that poor housing and unemployment were linked to increased A&E attendance rates [19], whereas those from an ethnic minority background had a 39% lower risk of attending A&E [20].

## Discussion

This study reports the first overarching findings of the longitudinal NWC HHS, which explores health inequalities in accessing health and social care services in some of the most disadvantaged neighbourhoods in the country.

This longitudinal survey captures a broad range of variables ranging from mental and physical health to socioeconomic factors in some of the most socio-economically disadvantaged areas of England [13]. The design process of this survey was unique in that a range of stakeholders contributed to the survey development, including researchers, local authority partners, NHS partners and to a more limited extent, members of the public. The in-depth and collaborative design process and the subsequent conduct of the survey were strongly supported by the collaborative structure of the lead research organisation, the CLAHRC NWC. The very foundation of the CLAHRC is collaboration between researchers, health professionals, and other partner organisations, thereby facilitating co-produced research and building capacity in non-research partners. Co-production has been shown in other health research to be beneficial because it allows the experiences of people with a condition and trained staff to shape services and research [21, 22]. Similarly, here, we found that different perspectives improved the quality of the research and its dissemination, whilst being mindful of limited co-production in the design and implementation stages with some local authorities and the potential impact on lower survey response rates in those areas. This could be addressed by more active co-production in future in the very early stages.

Comparing the survey data to national data, the present sample was biased towards female respondents (our sample: 57.1%, census: 50.9%) [23] and Black and Minority ethnic participants (our sample: 11%, census: 8%) compared to census data for North West England [24]. However, ethnicity and gender did not vary as a function of disadvantage. People in more disadvantaged neighbourhoods were younger than people in relatively advantaged neighbourhoods. Taken together, the neighbourhood types were well matched demographically and were slightly biased on gender and ethnicity compared to census statistics.

Looking at the variations between more and relatively advantaged neighbourhoods, socioeconomic factors differed between neighbourhoods in the expected directions, with less employment, lower education, and higher proportions of social housing in disadvantaged areas. Social housing, however, was only found to be higher in the NfLs, but not in the comparator sites. General health status was better in the relatively advantaged areas, but health seemed to be worse in the NfLs compared to the disadvantaged comparator neighbourhoods. Nevertheless, of the healthcare utilization variables examined, only A&E attendance was found to be higher in disadvantaged neighbourhoods compared to the relatively advantaged areas, whilst GP attendance rates did not differ between neighbourhoods. Comparing this to national data, A&E attendance in our survey (20–27%) was lower than the 36% reported at the national level in 2017. GP attendance in our survey (69–70%) was also lower than figures reported in the GP patient survey, which reported 83.4% of respondents having attended a GP in the previous 12 months [25]. Divergent findings on A&E attendance may be due to the nature of our sampling which involved recall over the previous 12 months as opposed to hospital data at time of attendance. The two GP surveys were both self-report, so the reason for this discrepancy is less clear. One possibility is that people who attend GPs more frequently are more likely to complete a GP patient survey. Our survey, on the other hand, may not be subject to this specific bias.

Whilst the survey collected data from a wide geographical area from both disadvantaged and relatively advantaged areas, there were some limitations that should be considered. For example, interviewers ensured to knock on residents’ houses during different times of the day, but evening sampling was limited due to practical constraints. Because of this, people in full-time employment who were at work all day are likely to be underrepresented in our sample. Considering the focus on people living in disadvantaged neighbourhoods, data are limited to those with a fixed address. Thus, the survey was not able to capture some of the most disadvantaged groups in the population, such as homeless people and unregistered migrants. In addition, because specific neighbourhoods were selected for recruitment, this survey does not comprise a random sample of the general population. However, neighbourhoods were purposefully selected based on their level of deprivation, therefore providing a suitable sample for the focus of this survey. In addition, there were health and demographic differences between people who participated in both waves of the survey and those who did not, insofar as people who dropped out were more likely to be male, in paid employment, and not have a long-term health condition. This has implications for future recruitment strategies, which may benefit from extra measures to reduce drop-out among specific groups, such as instigating higher number call-backs before terminating follow-up or adjusting call-back times to increase retention of people in paid employment.

One of the lessons learned from designing, setting up, conducting, and analysing this survey is the benefit of having co-produced the survey with partners. Involving partners at every step of the process has helped to guide the research and outputs, but also to interpret findings and contribute to outputs from a non-researcher point of view. However, partner involvement was only very small at the beginning, and needs to be amplified further in future steps. Members of the public have been particularly involved in the current dissemination of the findings [26], including in this write up (TW, KH), and have helped shape the planning for Wave 2.

## Conclusions

The NWC HHS has already highlighted several inequalities in accessing health care services, and is one of the first longitudinal public health surveys across England to specifically focus on people living in some of the most disadvantaged neighbourhoods in the country. Findings can help identify key areas of needs to tackle to reduce health inequalities, thereby addressing the World Health Organisation’s recent Health Equity Status Report [23] and providing guidance for how to address one of the five essential conditions for healthy lives for everyone: “good quality and accessible health services” [5].

## Supplementary information

**Additional file 1.** The CLAHRC NWC Household Health Survey. Public health survey.

## Data Availability

Users can obtain access to the ARC NWC HHS data files after submitting a brief proposal (including agreement to HHS’ conditions of use) at [info@pldr.org]. Users will also be required to outline which version of the survey dataset they wish to access, data security arrangements in place and how they meet the criteria for access. Access to the data will be authorized following approval from the PLDR governance board.

## Abbreviations

HHS: Household Health Survey
HIAT: Health Inequalities Assessment Toolkit
NWC: North West Coast
SES: Socio-economic status
