# Supplementary material for: The longitudinal NIHR ARC North West Coast Household Health Survey: exploring health inequalities in disadvantaged communities
Source: BMC Public Health. 2020 Aug 18;20:1257. doi: 10.1186/s12889-020-09346-5 (PMC7436975; doi:10.1186/s12889-020-09346-5)
Supplement: Supplementary file 1 — Additional file 1. The CLAHRC NWC Household Health Survey. Public health survey. [file 12889_2020_9346_MOESM1_ESM.pdf]

The CLAHRC NWC
Household Health Survey

Good morning \ afternoon, my name is … and I'm from BMG Research.

BMG Research is an independent social research company who work to the Market Research Society (MRS) code of conduct. We are carrying out research on behalf of the University of Liverpool, University of Central Lancashire and the University of Lancaster as part of the NHS National Institute of Health Research Collaboration for Leadership in Applied Health Research and Care Programme North West Coast.

You should have received a letter and leaflet about this research in the past few weeks [SHOW LAMINATED LETTER & INFORMATION LEAFLET]. The survey is intended to contribute to the development of action to improve health and wellbeing particularly in those neighbourhoods that have the poorest indicators of health. It explores such aspects as general health, wellbeing and mental health, views of your local area, and community engagement.

The interview will take approximately 45 minutes, and you will receive a £10 gift voucher as a thank you for taking part.

IF LONGITUNAL NAMED CONTACT
QA. In one of our earlier surveys, we interviewed (NAME OF PREVIOUS PARTICIPANT) from this address. Can we speak to (NAMED CONTACT)?

Interviewer note: If person asks 'why' or 'why do you need to speak to (NAMED CONTACT) rather than me', state: **We would most like to talk to people we have interviewed before so that we can examine how their own circumstances and opinions have changed over time. It allows us to draw the most accurate conclusions from the study. As such, we would like to speak to (NAMED CONTACT) as a priority in the first instance.**

1	Yes, I am (NAMED CONTACT)	1 (**GO TO DATA ASSURANCE TEXT)**
2	Yes, but I am not (NAMED CONTACT)	2 (**GO TO INTERVIEWER INSTRUCTIONS A**)
3	No, that person is no longer a member of this household	3 FIND OUT IF A CURRENT HOUSEHOLDER IS 18+ AND IS WILLING TO TAKE PART
4	No, that person has never been a member of this household	4 FIND OUT IF A CURRENT HOUSEHOLDER IS 18+ AND IS WILLING TO TAKE PART
5	Don't know	4 FIND OUT IF A CURRENT HOUSEHOLDER IS 18+ AND IS WILLING TO TAKE PART

INTERVIEWER INSTRUCTIONS A:
ASK TO SPEAK TO NAMED CONTACT (PREVIOUS SUREVY PARTICIPANT).

i. IF PERSON IS AVAILABLE, **DATA ASSURANCE TEXT**
ii. IF PERSON IS NOT AVAILABLE, FIND OUT A TIME FOR A REPEAT VISIT.
iii. IF YOU ARE TOLD THAT AN INTERVIEW WITH THE PREVIOUS PARTICIPANT IS NOT POSSIBLE AT ALL, ASK WHETHER ANY ELIGIBLE OTHER HOUSEHOLDERS WOULD BE WILLING TO TAKE PART

Data Assurance Text

BMG Research will only use your details for the purpose of the NIHR CLAHRC NWC's research , and for quality checking the interviews. The anonymised findings from the survey may be published. BMG Research abides by the Market Research Society Code of Conduct at all times.

You can find out more information about our surveys and what we do with the information we collect in our Privacy Notice which is on our website. You can find the details here INTERVIEWER TO HIGHLIGHT RELEVANT SECTION ON INFORMATION SHEET.

If you change your mind and wish to have the responses you give and your personal details deleted you can do so at any time, please just say. If you decide to do this after you have completed the survey you can find out how to do so in our Privacy Notice

IF RESPONDENT REQUIRES FURTHER CLARIFICATION THAT BMG RESEARCH IS A GENUINE MARKET RESEARCH COMPANY THEY CAN CALL MRS ON 0500 39 69 99.

INTERVIEWER NOTE: RESPONDENT MUST BE AGED 18 OR OVER

BEFORE PROCEEDING TO CONSENT FORM, INTERVIEWER TO SUMMARISE TYPE OF RESPONDENT BEING INTERVIEWED:

Same respondent as interviewed at earlier survey wave (named contact)	1
Same household as interviewed at earlier survey wave, but not a named contact (previous participant)	2
Neither respondent nor household interviewed at earlier wave	3

SCRIPTING INSTRUCTION: ADD A HIDDEN 'REFUSED' OPTION TO EVERY QUESTION (where there isn't one already included as part of the main question)

INTERVIEWER: ASK RESPONDENT TO READ RESEARCH CONSENT FORM AND SIGN CONTACT SHEET FOR SIGNATURE TO BE CAPTURED ELECTRONICALLY ON THE CAPI UNIT

PARTICIPANT CONSENT FORM

NWC CLAHRC HOUSEHOLD HEALTH SURVEY

1. I confirm that I have read and have understood the information sheet for the above study. I have had the opportunity to consider the information, ask questions and have had these answered satisfactorily.

2. I understand that my participation is voluntary and that I am free to withdraw at any time without giving any reason, without my rights being affected. In addition, should I not wish to answer any particular question or questions, I am free to decline.

3. I understand that, under the General Data Protection Regulation (2018) , I can at any time ask for any data I provide that could be used to identify me, to be destroyed.

4. I agree to take part in the above study.

______________________________	__________	____________________
Participant Name	Date	Signature

Section 1: About you and your household

These questions explore some details about you and your household. We recognise that you might consider some of these questions to be personal or sensitive, in which case you are free not to answer them. The information you provide will be used for the sole purpose of improving health and wellbeing, particularly in those neighbourhoods that have the poorest indicators of health.

(SOURCE: ONS)

1. I would first like you to think of everyone living in your household, including any children. How many people live here regularly as members of this household? Please include yourself in this figure. **WRITE IN BELOW ALL LIST**

COMPLETE GRID FOR EACH PERSON IN THE HOUSEHOLD. COLLECT NAME/ INITIALS OF EACH HOUSEHOLD MEMBER TO ENABLE PROMPTS.

(GRID for Q1)

a	**How many people live here including you?** CIRCLE AS APPROPRIATE<br><br>**NAME/INITIALS**	**1 Self**	**2**	**3**	**4**	**5**	**6**	**7**	**8**	**9**
b	RECORD GENDER. **And is ... male or female? ALL S/C**									
	Male	1	1	1	1	1	1	1	1	1
	Female	2	2	2	2	2	2	2	2	2
	Other	3	3	3	3	3	3	3	3	3
c	**What was your age last birthday? (What was [name's] age last birthday?) ALL LIST**									
c	**IF REFUSED, SHOWCARD 1: Which age band do you fall into? (Which age band does [name] fall into?) Q1c/REF S/C**									
	Under 16 years	-	1	1	1	1	1	1	1	1
	16-17 years	-	2	2	2	2	2	2	2	2
	18-24 years	3	3	3	3	3	3	3	3	3
	25-34 years	4	4	4	4	4	4	4	4	4
	35-44 years	5	5	5	5	5	5	5	5	5
	45-54 years	6	6	6	6	6	6	6	6	6
	55-64 years	7	7	7	7	7	7	7	7	7
	65-74 years	8	8	8	8	8	8	8	8	8
	75 years and over	9	9	9	9	9	9	9	9	9
	Refused	10	10	10	10	10	10	10	10	10
d	**What is the relationship of each household member to you? They are my...** ONE CODE ONLY PER HH MEMBER **ALL OTHER HOUSEHOLD MEMBERS S/C**									
	Spouse/civil partner/cohabiting partner	-	1	1	1	1	1	1	1	1
	Son/daughter (including step/ adopted/ foster/ in law)	-	2	2	2	2	2	2	2	2
	Grandson/granddaughter (including step/adopted)	-	3	3	3	3	3	3	3	3
	Parent/parent in-law/step-parent/guardian	-	4	4	4	4	4	4	4	4
	Brother/sister (including step/ adopted/ foster/ in-law)	-	5	5	5	5	5	5	5	5
	Grandparent	-	6	6	6	6	6	6	6	6

	Other relative	-	7	7	7	7	7	7	7	7
	Other non-relative	-	8	8	8	8	8	8	8	8
	REFUSED (DO NOT READ OUT)	-	9	9	9	9	9	9	9	9
e	**SHOWCARD 2. Which of these best describes your / (name's) current position?** ASK FOR ALL IN HH AGED 16+ ACCEPT ONLY ONE ANSWER PER HH MEMBER. **Q1c/16+ S/C**									
	Going to school or college full time (including on vacation)	1	1	1	1	1	1	1	1	1
	In paid employment or self employed (or temporarily away)	2	2	2	2	2	2	2	2	2
	On a Government scheme for employment training	3	3	3	3	3	3	3	3	3
	Doing unpaid work for a business that you own, or that a relative owns	4	4	4	4	4	4	4	4	4
	Waiting to take up paid work already obtained	5	5	5	5	5	5	5	5	5
	Looking for paid work or a Government training scheme	6	6	6	6	6	6	6	6	6
	Intending to look for work but prevented by temporary sickness or injury	7	7	7	7	7	7	7	7	7
	Permanently unable to work because of long-term sickness or disability	8	8	8	8	8	8	8	8	8
	Retired from paid work	9	9	9	9	9	9	9	9	9
	Looking after the home or family	10	10	10	10	10	10	10	10	10
	Doing something else, specify LIST (SENSE CHECK TO FIT IN AN ABOVE CODE)	95	95	95	95	95	95	95	95	95
	REFUSED (DO NOT READ OUT)	98	98	98	98	98	98	98	98	98

INTERVIEWER READ OUT: Now thinking about yourself...

2. What is your ethnic group? Please choose one option that best describes your ethnic group or background. **SHOWCARD 3 AND CODE ONE ONLY** (SOURCE: ONS) **ALL S/C**

	**White**
1	English / Welsh / Scottish / Northern Irish / British
2	Irish
3	Gypsy or Irish Traveller
4	Any other White background, *please specify*
	**Mixed / Multiple ethnic groups**
5	White and Black Caribbean
6	White and Black African
7	White and Asian
8	Any other Mixed / Multiple ethnic background, *please specify*
	**Asian / Asian British**
9	Indian
10	Pakistani
11	Bangladeshi
12	Chinese
13	Any other Asian background, *please specify*
	**Black / African / Caribbean / Black British**
14	African
15	Caribbean
16	Any other Black / African / Caribbean background, *please specify*
	**Other ethnic group**

17	Arab
95	Any other ethnic group, *please specify*<br>LIST (SENSE CHECK TO FIT IN AN ABOVE CODE)
98	Prefer not to say

3. What is your religion or belief? **SHOWCARD 4 AND CODE ONE ONLY** (SOURCE: HSE) **ALL S/C**

1	No religion
2	Christian – Catholic
3	Christian – all other denominations including Church of England, Protestant
4	Buddhist
5	Hindu
6	Jewish
7	Muslim
8	Sikh
95	Any other religion (please specify)<br>**LIST** (SENSE CHECK TO FIT IN AN ABOVE CODE)
98	Prefer not to say

4. To what extent do you agree or disagree that your personal religious beliefs or faith are important to you? **SHOWCARD 5 AND CODE ONE ONLY ALL S/C**

Strongly agree	1
Tend to agree	2
Neither agree nor disagree	3
Tend to disagree	4
Strongly disagree	5
Not applicable	6

5. Please can you tell me your marital or same-sex civil partnership status? Are you ...? **SHOWCARD 6 AND CODE ONE ONLY** (SOURCE: ONS) **ALL S/C**

Single, that is, never married and never registered in a same-sex civil partnership	1
Married	2
Separated, but still legally married	3
Divorced	4
Widowed	5
In a registered same-sex civil partnership	6
Separated, but still legally in a same-sex civil partnership	7
Formerly in a same-sex civil partnership which is now legally dissolved	8
Surviving partner from a same-sex civil partnership	9
Prefer not to say	10

6. Do you have any educational qualifications for which you received a certificate? **CODE ONE ONLY** (SOURCE: ONS) **ALL S/C**

Yes	1
No	2

7. Do you have any professional, vocational or other work-related qualifications for which you received a certificate? **CODE ONE ONLY** (SOURCE: ONS) **ALL S/C**

Yes	1
No	2

ASK Q8 WHERE CODED 1 AT Q6 OR Q7

8. What is your highest qualification? **READ OUT AND CODE ONE ONLY** (SOURCE: ONS) **Q6/1 OR Q7/1 S/C**

At degree level or above	1
Another kind of qualification	2

ASK ALL

9. Do you look after, or give any help or support to family members, friends, neighbours or others because of either a long term physical or mental ill-health / disability or problems related to old age? Do not count anything you do as part of your paid employment. **SHOWCARD 6B READ OUT AND CODE ONE ONLY** (SOURCE: CENSUS) **ALL S/C**

No	1
Yes, 1-19 hours per week	2
Yes, 20-49 hours per week	3
Yes, 50 or more hours per week	4

10. Which of the options [on this card] best describes how you think of yourself? Please just read out the number next to the description. **SHOWCARD 7 AND CODE ONE ONLY** (SOURCE: ONS) **ALL S/C**

Heterosexual or straight	1
Gay or lesbian	2
Bisexual	3
Other	4

Section 2: Housing

Now thinking about the house where you live...

11. In which of these ways does your household occupy this accommodation? **SHOWCARD 8 AND CODE ONE ONLY** (SOURCE: HSE) **ALL S/C**

Own it outright	1
Buying it with the help of a mortgage or loan	2
Part rent and part mortgage (shared ownership)	3
Rent it	4
Live here rent-free (incl. rent-free in relative's/friend's property excluding squatting)	5
Squatting	6
Other	7

ASK Q12 IF CODED 3 OR 4 AT Q11

12. Who is your landlord? SHOWCARD 9 AND CODE ONE ONLY (SOURCE: HSE) Q11/3-4 S/C

The local authority/council	1
A housing association or co-operative or charitable trust or registered social landlord	2
Employer (organisation) of a household member	3
Another organisation	4
Relative/friend (before you lived here) of a household member	5
Employer (individual) of a household member	6
Letting agency or another individual private landlord	7
Other	8

ASK ALL

13. How long have you lived at this address? **CODE ONE ONLY** (SOURCE: EHS) **ALL S/C**

Less than 12 months	1
12 months but less than 2 years	2
2 years but less than 3 years	3
3 years but less than 5 years	4
5 years but less than 10 years	5
10 years or more	6
Don't know	7

14. How many bedrooms does your household have, including bedsitting rooms and spare bedrooms? EXCLUDE BEDROOMS CONVERTED TO OTHER USES (E.G. BATHROOM). INCLUDE BEDROOMS TEMPORARILY USED FOR OTHER THINGS (E.G. STUDY, PLAYROOM) (SOURCE: HSE) **ALL LIST**

WRITE NUMBER IN THE BOX: | | |

15. During the winter months, does condensation form on the windows or walls of any room in your home apart from the bathrooms or toilets? **CODE ONE ONLY** (SOURCE: EHS) **ALL S/C**

Yes	1
No	2
Spontaneous: Don't know	3

16. During the winter months, are there patches of mould or fungus in any room in your home, apart from bathrooms or toilets? **CODE ONE ONLY** (SOURCE: EHS) **ALL S/C**

Yes	1
No	2
Spontaneous: Don't know	3

17. During the cold winter weather, can you normally keep comfortably warm in your living room? **CODE ONE ONLY** (SOURCE: EHS) **ALL S/C**

Yes	1
No	2
Spontaneous: Don't know	3

ASK Q18 IF CODED 2 AT Q17

18. Is this because...? READ OUT AND CODE ONE ONLY (SOURCE: EHS) Q17/2 S/C

It costs too much to keep your heating on	1
It is not possible to heat the room to a comfortable standard (for example, heating equipment is broken or under repair)	2
Both of the above (spontaneous only)	3
Neither	4
Not applicable	5
Don't know (spontaneous only)	6

ASK Q19a-19e) WHERE CODED 3 OR 4 AT Q11 (HAS A LANDLORD)

19. Overall, how satisfied or dissatisfied are you with the way your [Landlord] repairs and maintains your home? **SHOWCARD 10 AND CODE ONE ONLY** (SOURCE: EHS) **Q11/3-4 S/C**

Very satisfied	1
Fairly satisfied	2
Neither satisfied nor dissatisfied	3
Fairly dissatisfied	4
Very dissatisfied	5
Question not applicable (spontaneous only)	6

ASK Q19a IF CODED 4-5 AT Q19

19a What are the main reasons why you are dissatisfied? **DO NOT READ OUT. CODE ALL THAT APPLY.**

Landlord does not bother about repairs and maintenance	1
Landlord carries out only emergency repairs	2
Landlord is difficult to contact	3
Work done is of poor quality	4
Landlord does only the bare minimum	5
Landlord is slow to get things done	6
Landlord becomes hostile if maintenance issues are raised	7
Other (please specify)	8

Q19b On the whole, do you think that over the past two years the housing service provided by your landlord has got better or worse, or haven't things changed much? **SINGLE CODE**

Has got better	1
Has got worse	2
Has not changed much	3
I have been a tenat of this landlord for less than 2 years	4

ASK Q19C- ONLY FOR THE FOLLOWING NEIGHBOURHOODS: CLAREMONT WARD (BLACKPOOL), FAZARKELY (LIVERPOOL), OLD SWAN (LIVERPOOL)

Q19c Did you know that private landlords in your neighbourhood need to have a license from the council to rent properties? **SINGLE CODE**

Yes	1
No	2

19d. Do you know how to raise any concerns about the condition of your home with your landlord? (e.g faulty electrics, needing repairs, problems with security)? **SINGLE CODE**

Yes	1
No	2

Q19e Do you know how to make a complaint about your landlord to the council? **SINGLE CODE**

Yes	1
No	2

[ONLY ASK 19F IN CLAREMONT WARD (BLACKPOOL).

19f. Which of the following sources of advice and support have you used in the last year. **SHOWCARD. CODE ALL MENTIONED**

Egerton Road Office	1
Claremont Community Centre	2
ASB officer	3
Children's Centre	4
The Council's Web site	5
Fylde Coast Information Web site	6
First Steps	7
Customer First (Council office ring or call in)	8
Age UK	9
Housing Options – South King street	10
A Housing Association	11
Horizon	12
The Peoples Team	13
Social Services	14
GP	15
MP	16
Ward Councillor	17
Church	18
Citizens Advice Bureau	19

Section 3: Family and friends

Now thinking about your family and friends...

ASK ALL

20. Not counting the people you live with, how often do you meet up in person with family members or friends?

REMIND IF NECESSARY: Not counting the people you live with. **READ OUT AND CODE ONE ONLY** (SOURCE: COMMUNITY LIFE SURVEY) **ALL S/C**

More than once a day	1
Once a day	2
4-6 times per week	3
2-3 times per week	4
About once a week	5
About once a fortnight	6
About once a month	7
Less often than once a month	8
Never	9

21. How much do you agree or disagree with the following statements? **SHOWCARD 11 AND CODE ONE FOR EACH** (SOURCE: COMMUNITY LIFE SURVEY) **ALL S/C PER ROW**

	Definitely agree	Tend to agree	Tend to disagree	Definitely disagree	Don't know
If I needed help, there are people who would be there for me	1	2	3	4	5
If I wanted company or to socialise, there are people I can call on	1	2	3	4	5

INTERVIEWER NOTE THAT THE SCALE HAS REVERSED ROUND, WITH STRONGLY DISAGREE SHOWING FIRST

22. Here is a list of statements about how you feel about yourself **RIGHT NOW**. Please consider each one carefully, and indicate to what extent you agree or disagree? **SHOWCARD 12**, **READ OUT STATEMENTS AND CODE ONE PER ROW ALL S/C PER ROW**

	Strongly disagree	Disagree	Neither agree or disagree	Agree	Strongly agree
The future seems to me to be hopeful and I believe that things are changing for the better	1	2	3	4	5
I feel that it is possible to reach the goals I would like to strive for	1	2	3	4	5

23. To what extent do you agree or disagree with each of the following statements? **SHOWCARD 12, READ OUT STATEMENTS AND CODE ONE PER ROW ALL S/C PER ROW**

	Strongly disagree	Disagree	Neither agree or disagree	Agree	Strongly agree
Often there is no chance of protecting my personal interest from bad luck happenings	1	2	3	4	5
My life is determined by my own actions	1	2	3	4	5
I am usually able to protect my personal interests	1	2	3	4	5
I feel like what happens in my life is mostly determined by powerful people	1	2	3	4	5
To a great extent my life is controlled by accidental happenings	1	2	3	4	5
When I get what I want, it is usually because I am lucky	1	2	3	4	5
People like myself have very little chance of protecting our personal interests when they conflict with those of strong pressure groups	1	2	3	4	5
I can pretty much determine what will happen in my life	1	2	3	4	5
My life is chiefly controlled by powerful others	1	2	3	4	5

Section 4: Neighbourhood

INTERVIEWER READ OUT: Now I would like to ask you some questions about your immediate neighbourhood, by which I mean your street or block.

Q24a Thinking about your immedidate neighbourhood, to what extent do you agree or with these statements? **SHOWCARD, READ OUT STATEMENTS AND CODE ONE PER ROW**

	Strongly disagree	Disagree	Neither agree or disagree	Agree	Strongly agree
I identify with my local neighbourhood	1	2	3	4	5
I feel committed to my local neighbourhood	1	2	3	4	5
I am glad to be part of my local neighbourhood	1	2	3	4	5
Being part of my local neighbourhood is an important part of how I see myself	1	2	3	4	5

24. Using the answers on this card, please tell me how strongly you feel you belong to your immediate neighbourhood? **SHOWCARD 13 AND CODE ONE ONLY** (SOURCE: COMMUNITY LIFE / CITIZENSHIP SURVEY) **ALL S/C**

Very strongly	1
Fairly strongly	2
Not very strongly	3
Not at all strongly	4
Don't know	5

25. How comfortable would you be with asking a neighbour to keep a set of keys to your home for emergencies, for example if you were locked out. **SHOWCARD 14 CODE ONE**

Very comfortable	1
Fairly comfortable	2
Fairly uncomfortable	3
Very uncomfortable	4

26. Suppose you lost your purse/wallet containing your address details, and it was found in the street by someone living in this neighbourhood. How likely is it that it would be returned to you with nothing missing? **SHOWCARD 15 AND CODE ONE ONLY** (SOURCE: ONS CAPITAL HARMONIZED QUESTIONS / 2004 GHS) **ALL S/C**

Very likely	1
Quite likely	2
Not very likely	3
Not at all likely	4
Don't know	5

27. Would you say this neighbourhood is a place where neighbours look out for each other? **READ OUT AND CODE ONE ONLY** (SOURCE: CITIZENSHIP SURVEY) **ALL S/C**

Yes, definitely	1
Yes, to some extent	2
No	3
Don't know	4
Not stated	5

28. I am going to read out a list of problems which some people face in their neighbourhood. For each one, please can you tell me how much of a problem it is in your neighbourhood? **SHOWCARD 16 AND CODE ONE PER ROW** (SOURCE: ONS CAPITAL HARMONIZED QUESTIONS) **ALL S/C PER ROW**

	Very big problem	Fairly big problem	Not a very big problem	Not a problem at all	It happens but it's not a problem	Don't know
People being drunk or rowdy in public places	1	2	3	4	5	6
Rubbish or litter lying around	1	2	3	4	5	6
Vandalism, graffiti and other deliberate damage to property or vehicles	1	2	3	4	5	6
People being attacked or harassed because of their skin colour, ethnic origin or religion	1	2	3	4	5	6
Teenagers hanging around on the street	1	2	3	4	5	6
Troublesome neighbours	1	2	3	4	5	6
The speed or volume of road traffic	1	2	3	4	5	6
Traffic exhaust fumes	1	2	3	4	5	6

29. When did you last use or visit each of the following? Please don't include any visits you made whilst on holiday or otherwise away from home for more than a day. **SHOWCARD 17 AND CODE ONE PER ROW ALL S/C PER ROW**

	Yester-day	Within the last week	Within the last month	Within the last 6 months	Within the last year	Longer ago	Never	N/A	Don't know / can't remember
A park in a town or city	1	2	3	4	5	6	7	8	9
An allotment or community garden	1	2	3	4	5	6	7	8	9

A children's playground	1	2	3	4	5	6	7	8	9
A playing field or other recreation area	1	2	3	4	5	6	7	8	9
Open countryside (e.g. woodland, farmland, a mountain, hill or moorland, river, lake, or canal)	1	2	3	4	5	6	7	8	9
A path, cycleway or bridleway	1	2	3	4	5	6	7	8	9
Country park	1	2	3	4	5	6	7	8	9
Coast/beach areas	1	2	3	4	5	6	7	8	9
A local community centre	1	2	3	4	5	6	7	8	9
A local leisure centre, pool or gym'.	1	2	3	4	5	6	7	8	9

28A. Here is a list of local services or organisations. Would you say they work in the best interests of the people in your neighbourhood… **SHOWCARD AND CODE ONE PER ROW**

	All of the time	Most of the time	Some of the time	Rarely	Never	Don't Know
Your local GP	1	2	3	4	5	6
The council	1	2	3	4	5	6
Your local hospital	1	2	3	4	5	6
Local charities or voluntary organisations	1	2	3	4	5	6
Local businesses	1	2	3	4	5	6

30. A) In which ways do you usually access the internet for your own use? **SHOWCARD 18 AND CODE ALL THAT APPLY** (SOURCE: GOWELL) **ALL M/C**

Mobile phone via wifi	1
Mobile phone via 3G or 4G	2
At home, on a desktop or laptop computer or tablet	3
Computer at work, school, college or university	4
Internet cafe, shop or public premises with wifi	5
Public library or community centre	6
At a friend's or other family member's house	7
Other	8
I don't have access to the Internet	9
I am unable to use the Internet	10

The next few questions relate to the social contact you have with people from other ethnic groups. This social contact may be positive (e.g., a friendly chat), negative (e.g., an argument), or neutral (e.g., buying an item from a shopkeeper). Please respond to the following questions about how much positive, negative and neutral contact you have with people from different ethnic groups to yours.

30c. On average, how frequently do you have POSITIVE/GOOD contact with people from other ethnic groups?

Never	Very rarely	Rarely	Sometimes	Quite Often	Often	Extremely frequently/ often
1	2	3	4	5	6	7

30d. On average, how frequently do you have NEGATIVE/BAD contact with people from other ethnic groups?

Never	Very rarely	Rarely	Sometimes	Quite Often	Often	Extremely frequently/ often
1	2	3	4	5	6	7

30e. On average, how often do you have CASUAL/INCIDENTAL/NEUTRAL contact with people from other ethnic groups?

Never	Very rarely	Rarely	Sometimes	Quite Often	Often	Extremely frequently/ often
1	2	3	4	5	6	7

Air pollution.

The next few questions are about air quality in your neighbourhood?

Q30f How would you rate the overall air quality in your neighbourhood now compared to last year? **SHOWCARD . SINGLE CODE**

Much better	1
A little better	2
About the same	3
A little worse	4
Much worse	5

30g. To what extent does air pollution in your neighbourhood affecting you? **SHOWCARD . SINGLE CODE**

Very much	1
A little	2
Not at all	3

ASK IF CODED 1 OR 2 AT Q30G

30h. In which of the following ways are you affected? **SHOWCARD. MULTICODE**

Breathlessness/having more difficulty in breathing	1
Irritation to your skin	2
Irritation to eyes/nose/throat	3
Feeling depressed	4
Poor visibility	5
Bad smells	6
I do less outdoor activity becuse of air quality	7
I want to move to a less polluted place	8
I worry about the living environment for me /my family	9
Air quality where I live does not have any of these impacts	10

Section 5: Civic engagement

Now thinking about whether you can influence political decisions and local affairs. These questions refer to your local area, which is within 15 to 20 minutes walk from here.

31. In the last 12 months, that is since [INSERT DATE ONE YEAR AGO], have you done any of the following? **SHOWCARD 19 AND CODE ALL THAT APPLY** (SOURCE: COMMUNITY LIFE SURVEY) **ALL M/C**

Contacted a local official such as a local councillor, MP, government official, mayor, or public official working for the local council. PLEASE EXCLUDE CONTACT FOR PERSONAL ISSUES SUCH AS HOUSING REPAIRS OR CONTACT THROUGH EMPLOYMENT	1
Attended a public meeting or rally, taken part in a public demonstration or protest	2
Signed a paper petition or an online/e-petition	3
None of the above	4

32. In the last 12 months, that is since [INSERT DATE ONE YEAR AGO], have you done any of the following? **SHOWCARD 20 AND CODE ALL THAT APPLY** (SOURCE: COMMUNITY LIFE SURVEY) **ALL M/C**

Taken part in a consultation about local services or problems in your local area through completing a paper on online questionnaire	1
Taken part in a consultation about local services or problems in your local area through attending a public meeting.	2
Taken part in a consultation about local services or problems in your local area through being involved in a face-to-face or online group.	3
None of these	4

33. And again in the last 12 months, that is since [INSERT DATE ONE YEAR AGO], have you been a member of any of the groups listed on this card? Please include online groups and any activities you have already told me about. Do not include any activities related to your job. **SHOWCARD 21 AND CODE ALL THAT APPLY** (SOURCE: COMMUNITY LIFE SURVEY) **ALL M/C**

A group making decisions on local health services	1
A decision making group set up to regenerate the local area	2
A decision making group set up to tackle local crime problems	3
A tenants' group decision making committee	4
A group making decisions on local education services	5

A group making decisions on local services for young people	6
Another group making decisions on services in the local community	7
None of these	8

34. Do you agree or disagree that you can influence decisions affecting your local area? Just a reminder that your local area is within a 15 to 20 minute walk from here. **SHOWCARD 22 AND CODE ONE ONLY** (SOURCE: COMMUNITY LIFE SURVEY) **ALL S/C**

Definitely agree	1
Tend to agree	2
Tend to disagree	3
Definitely disagree	4
Don't know	5

35. Thinking about the last time there was a local government election, did you vote? This does not include the election of the local police and crime commissioners. **CODE ONE ONLY ALL S/C**

Yes	1
No	2
Not eligible to vote	3
Don’t know	4

36. Please select from the following which best describes any groups, clubs or organisations you've taken part in, supported or helped, over the last 12 months. **SHOWCARD 23 AND CODE AS MANY AS APPLY** (SOURCE: CITIZENSHIP SURVEY) **ALL M/C**

Children's education/ schools	1
Youth/children's activities (outside school)	2
Education for adults	3
Sports/exercise (taking part, coaching or going to watch)	4
Religion (e.g. attending a church, mosque or other place of worship)	5
Politics (e.g. being involved with a political party)	6
The elderly	7
Health, Disability and Social welfare	8
Safety, First Aid	9
The environment, animals	10
Justice and Human Rights	11
Local community or neighbourhood groups	12

Citizens' Groups	13
Hobbies / Recreation / Social clubs	14
Trade union activity	15
Arts (i.e. painting groups, drama, or other arts activities)	16
Musical group	17
Other (please specify)…**LIST** (SENSE CHECK TO FIT IN AN ABOVE CODE)……………………	95
None of these	96

ASK Q37 WHERE CODED 1-95 AT Q36

37. Now I would like you to look at this showcard. In the last 12 months have you given unpaid help to any groups, clubs or organisations in any of the ways shown on this card? **SHOWCARD 24 AND CODE AS MANY AS APPLY** (SOURCE: CITIZENSHIP SURVEY) **Q36/1-95 M/C**

Raising or handling money/ taking part in sponsored events/ Leading the group/ member of a committee	1
Organising or helping to run an activity or event	2
Visiting people	3
Befriending or mentoring people	4
Giving advice/information/counselling	5
Secretarial, admin or clerical work	6
Providing transport/driving	7
Representing	8
Campaigning	9
Other practical help (e.g. helping out at school, shopping)	10
None of the above	11

ASK Q38 IF CODED 1-10 AT Q37

38. Overall, about how often over the last 12 months have you generally done something to help this/these group(s), club(s) or organisation(s). Would you say...? **READ OUT AND CODE ONE ONLY** (SOURCE: CITIZENSHIP SURVEY) **Q37/1-10 S/C**

At least once a week	1
Less than once a week but at least once a month	2
Less often	3
Other	4

Section 6: Work and financial security

This section will explore your current or past employment, as well as feelings of financial security.

ASK Q39 IF NOT IN EMPLOYMENT - Q1E (ALL BUT CODE 2)

39. Have you ever been in PAID employment or self-employed? **CODE ONE ONLY** (SOURCE: HSE) **Q1e/ALL BUT CODE 2, S/C**

Yes	1
No	2

ASK Q40 IF CODED 1 AT Q39

40. Which year did you leave your last PAID job? **WRITE IN YEAR BELOW (ESTIMATE IF UNSURE)** (SOURCE: HSE) **Q39/1 LIST**

ASK Q41-Q43 IF IN EMPLOYMENT – Q1E/2, OR CODED 1 AT Q39 AND WORKED IN LAST YEAR (Q40)

[The 3 questions below collect information for coding to the Standard Occupational Classification 2010 (SOC 2010). They ask about current job for those in paid work or about last main job for those who have ever had paid work, with the exception of full-time students and those who have been unemployed for more than a year who are allocated to residual categories.]

SCRIPTING INSTRUCTION: Can the past and present tense wording be filtered depending on whether the respondent is currently in employment (QE/1) or whether they are no longer in employment but have been employed in the past year (Q39/1 AND Q40=IN LAST YEAR).

41. What did/does the firm/organisation you worked/work for mainly make or do? **WRITE IN BELOW** (SOURCE: ONS)

DESCRIBE FULLY - PROBE MANUFACTURING OR PROCESSING OR DISTRIBUTING ETC., AND MAIN GOODS PRODUCED, MATERIALS USED, WHOLESALE OR RETAIL ETC. IT SHOULD BE NOTED THAT INFORMATION ON INDUSTRY IS NECESSARY TO DISTINGUISH BETWEEN SOME OCCUPATIONS AT THE DETAILED LEVEL.

LIST (CLEAN FOR TYPOS)<br><br>Don’t know 97

42. What was/is your (main) job? **WRITE IN** (SOURCE: ONS)

LIST (CLEAN FOR TYPOS)<br><br>Don’t know 97

43. Were/are you working as an employee or were/are you self-employed? **CODE ONE ONLY** (SOURCE: ONS)

Employee	1
Self-employed	2
Don't know	3

ASK Q44-45 IF CODED 1 AT Q43

44. In your job, did/do you have formal responsibility for supervising the work of other employees? **CODE ONE ONLY** (SOURCE: ONS) **Q43/1 S/C**

Yes	1
No	2

45. How many people worked/work for your employer at the place where you worked/work? **CODE ONE ONLY** (SOURCE: ONS) **Q43/1 S/C**

1-24	1
25-499	2
Or 500 or more employees	3
Don't know	4

ASK Q46 IF CODED 2 AT Q43

46. Were/are you working on your own or did/do you have employees? **CODE ONE ONLY** (SOURCE: ONS) **Q43/2 S/C**

On own / with partner(s) but no employees	1
With employees	2

ASK Q47 IF CODED 2 AT Q46

47. How many people did/do you employ at the place where you worked/work? **CODE ONE ONLY** (SOURCE: ONS) **Q46/2 S/C**

1-24	1
25-499	2
Or 500 or more employees	3

ASK Q48-50 IF IN EMPLOYMENT - Q1E/2

48. Are you working full-time or part-time? (Respondent's definition) **CODE ONE ONLY** (SOURCE: UNKNOWN, SIMILAR QUESTIONS INCLUDED IN LFS) **Q1e/2 S/C**

Full time	1
Part time	2

48N. How many jobs do you currently have? **WRITE IN BELOW**

49. How many hours per week do you usually work in your job/business (include all jobs if applicable)? **WRITE IN NUMBER IN HOURS BELOW, AND GET BEST ESTIMATE WHERE THEY ARE UNSURE** (SOURCE: adapted from LFS) **Q1e/2 LIST**

50. In your main job, does your employer guarantee a minimum set number of paid hours work each day/ week/ fortnight/ month? **CODE ONE ONLY** (SOURCE: ADAPTED FROM LFS CONSULTATION ON ZHC) **Q1e/2 S/C**

Yes	1
No	2

ASK Q51 IF CODED 1 AT Q50

51. How many hours are guaranteed? **WRITE IN NUMBER IN HOURS IN BOX BELOW** (SOURCE: ADAPTED FROM LFS CONSULTATION ON ZHC) **Q50/1 LIST**

ASK Q52 IF PROVIDED NUMBER OF HOURS AT Q51

52. Over what period? **WRITE IN** (SOURCE: ADAPTED FROM LFS CONSULTATION ON ZHC) **Q51 NOT REFS/C**

Per day	1
Per week	2
Per fortnight	3
Per month	4
Other (please specify) **LIST**	95
Don't know	97

ASK Q53-54 IF IN EMPLOYMENT - Q1E/2

53. Would you prefer to work longer hours at your current basic rate of pay – that is, not overtime or enhanced pay rates – if you were given the opportunity? **CODE ONE ONLY** (SOURCE: LFS) **Q1e/2 S/C**

Yes	1
No	2
Don't know	3

54. Will you please look at this card and tell me which group represents your usual take home pay from your current job(s) (include pay from all current jobs)? **SHOWCARD 24b AND CODE ONE ONLY** (SOURCE: LFS) **Q1e/2 LIST**

	Weekly			Monthly			Annually		
1	£0	-	£24	£0	-	£99	£0	-	£1,199
2	£25	-	£49	£100	-	£199	£1,200	-	£2,399
3	£50	-	£74	£200	-	£299	£2,400	-	£3,599
4	£75	-	£99	£300	-	£399	£3,600	-	£4,799
5	£100	-	£124	£400	-	£499	£4,800	-	£5,999
6	£125	-	£149	£500	-	£599	£6,000	-	£7,199
7	£150	-	£199	£600	-	£799	£7,200	-	£9,599
8	£200	-	£249	£800	-	£999	£9,600	-	£11,999
9	£250	-	£299	£1,000	-	£1,199	£12,000	-	£14,399
10	£300	-	£349	£1,200	-	£1,399	£14,400	-	£16,799
11	£350	-	£399	£1,400	-	£1,599	£16,800	-	£19,199
12	£400	-	£499	£1,600	-	£1,999	£19,200	-	£23,999
13	£500	-	£699	£2,000	-	£2,799	£24,000	-	£33,599
14	£700	-	£1,000	£2,800	-	£4,000	£33,600	-	£48,000
15	More than £1,000			More than £4,000			More than £48,000		
97	Don't know								
98	Prefer not to say								

ASK ALL

55. How well would you say your household is managing financially these days? Would you say you are...? **READ OUT AND CODE ONE ONLY** (SOURCE: WAS) **ALL S/C**

Doing well	1
Getting by	2
Struggling	3

56. Would you say your household is better off or worse off financially than you were a year ago? **READ OUT AND CODE ONE ONLY** (SOURCE: WAS) **ALL S/C**

Better off	1
About the same	2
Worse off	3
Don't know	4

ASK Q58 IF CODED 1 AT Q57

57. What are the main reasons for your household's financial situation getting better? **DO NOT PROMPT AND CODE AS MANY AS APPLY** (SOURCE: WAS) **Q57/1 M/C**

Increase in household income	1
Increase in welfare benefits	2
Receipt of lump sum payment (e.g. inheritance, gambling, redundancy, insurance or compensation claim payments)	3

Borrowed more money to make ends meet	4
Reduction in debt payments (inc. paid off debt)	5
Children no longer dependent (inc. left home)	6
General reduction in spending	7
Change in household circumstances (e.g. setting up home with a new partner or partner returning)	8
Reduction in caring responsibilities	9
Other reasons (please specify) **LIST**	95
Don't know	97

ASK Q59 IF CODED 3 AT Q57

58. What are the main reasons for your household's financial situation getting worse? **DO NOT PROMPT AND CODE AS MANY AS APPLY** (SOURCE: WAS) **Q57/3 M/C**

Reduction in household income	1
Reduction or loss of welfare benefits	2
Retired	3
Increased debt repayments	4
Increased spending on children in household	5
Unexpected or high bills	6
General increase in spending	7
Change in household circumstances (e.g. separation from partner / widowed)	8
Increased caring responsibilities	9
Losses from gambling or other speculation	10
Other reasons (please specify) **LIST**	95
Don't know	97

ASK ALL

59. I would now like to ask you about any debts, credit or loans you may have, apart from mortgages. Do you currently owe any money in any of the following ways? **SHOWCARD 25 AND CODE ALL THAT APPLY** (SOURCE: ADAPTED FROM UNDERSTANDING SOCIETY) **ALL M/C**

Credit Card	1
Hire Purchase (i.e. Brighthouse)	2
Payday lender	3
Pawn Shop (i.e. Cash Converter)	4
Local companies, including Moneyshop	5
Bank Overdraft	6
Fixed term loan from a Bank or Building Society (EXCLUDING a mortgage)	7
Loan from a Credit Union	8
Loan from a finance company	9
Loan from an unlicensed money lender	10
Loan from a friend or relative	11
Loan or advance on wages from your employer	12
Social Fund loan	13

Student Loans Company	14
Other (please specify)<br>LIST LIST (SENSE CHECK INTO AN ABOVE CODE)	95
None of these	96

ASK Q61 IF CODED 1-95 AT Q60

60. Have you been able to make the minimum payments on these loans or have you missed two or more consecutive payments in a row? **CODE ONE ONLY** (SOURCE: WAS) **Q60/1-95 S/C**

Able to make minimum payments	1
Missed two or more consecutive payments in a row	2
Refused	3
Not applicable	4

ASK Q62 IF CODED 1-95 AT Q60

61. About how much in total do you currently owe (i.e how much is left to be owed) on this or these commitments? **CODE ONE ONLY [ENCOURAGE AN ESTIMATE RATHER THAN CODING DON'T KNOW WHERE POSSIBLE]** (SOURCE: UNDERSTANDING SOCIETY) **Q60/1-95 S/C**

Less than £250	1
£250 to £499	2
£500 to £749	3
£750 to £999	4
£1,000 to £1,999	5
£2,000 to £2,999	6
£3,000 to £3,999	7
£4,000 to £4,999	8
£5,000 to £9,999	9
£10,000 or more	10
Don't know	11
Refused	12

[61A. -ONLY TO BE ASKED IN CENTRAL SOUTHPORT – DUKES AND CAMBRIDGE WARDS]

Q61a Which of the following sources of information have you used in the last 12 months to find out about anything to do with money – whether that is how to budget or plan your finances, the best insurance, banking or credit products available, how to claim benefits or grants, or to get any help or advice, etc. SHOWCARD. MULTICODE

1. Southport Debt Centre
2. Christians Against Poverty
3. 15. Citizens Advice Bureau
4. Southport Community Money Advice (Southport Food Bank)
5. Southport Food Pantry.
6. Migrant Workers Sefton Community (MWSC) charity
7. Sefton Advocacy Service
8. Southport One Stop Shop
9. The LIFE Rooms
10. Southport Community Centre.
11. One Vision Housing: Financial inclusion service.
12. Light for Life (Housing)
13. Sefton Credit Union
14. Friends
15. Family
16. Bank, building society or Insurance company
17. Professional financial adviser/planner
18. Other

[61a
ASK ALL

Q61b The following questions are about the food situation for your household in the past 12 months. I'm going to read you three statements that may be used to describe the food situation for a household. Please tell me if the statement was often true, sometimes true, or never true in the past 12 months**.SHOWCARD. SINGLE CODE FOR EACH**

	Often true	Sometimes true	Never true
You or your household have worried that food would run out before you got money to buy more.	1	2	3
The food that you/your houshold bought just didn't last, and there wasn't any money to get more.	1	2	3
You/ your household couldn't afford to eat balanced meals.	1	2	3

61c. How often have you used a food bank, or similar service, in the last year? **SINGLE CODE**

I have not used a food bank	1
At least weekly	2
About once or twice a month	3
Less than once a month	4
Don’t know	5
Prefer not to say	6

Section 7: Mental health and well-being

Now exploring your mental health and well-being.

INTERVIEWER: HAND THE CAPI UNIT TO THE RESPONDENT FOR THEM TO COMPLETE THESE QUESTIONS PRIVATELY.

ASK ALL

62. What best describes your experience of each of the following statements over the last two weeks? **SHOWCARD 26 AND CODE ONE PER ROW** (SOURCE: WARWICK-EDINBURGH MENTAL WELL-BEING SCALE, WEMWBS) **ALL S/C PER ROW**

	None of the time	Rarely	Some of the time	Often	All of the time
I've been feeling optimistic about the future	1	2	3	4	5
I've been feeling useful	1	2	3	4	5
I've been feeling relaxed	1	2	3	4	5
I've been dealing with problems well	1	2	3	4	5
I've been thinking clearly	1	2	3	4	5
I've been feeling close to other people	1	2	3	4	5
I've been able to make up my own mind about things	1	2	3	4	5

63. Over the last 2 weeks, how often have you been bothered by any of the following problems? **SHOWCARD 27 [if not self completing] AND CODE ONE PER ROW** (SOURCE: PHQ9) **ALL S/C PER ROW**

	Not at all	Several days	More than half the days	Nearly every day
Little interest or pleasure in doing things?	1	2	3	4
Feeling down, depressed, or hopeless?	1	2	3	4
Trouble falling or staying asleep, or sleeping too much?	1	2	3	4
Feeling tired or having little energy?	1	2	3	4
Poor appetite or overeating?	1	2	3	4
Feeling bad about yourself - or that you are a failure or have let yourself or your family down	1	2	3	4
Trouble concentrating on things, such as reading the newspaper or watching television?	1	2	3	4
Moving or speaking so slowly that other people could have noticed? Or the opposite - being so fidgety or restless that you have been moving around a lot more than usual?	1	2	3	4
Thoughts that you would be better off dead, or of hurting yourself in some way?	1	2	3	4

62b Have you deliberately hurt yourself in the past 12 months?

Yes	1
No	2
Prefer not to say	3

ASK 62C IF CODED 1 AT Q62B

Q62C Have you been to any of the following services because you deliberately hurt yourself?

	Yes	No
Been to a hospital casualty/A&E department?	1	2
Been admitted to hospital overnight as an in-patient?	1	2
Been to a health centre, GP surgery or practice?	1	2
Been seen by a GP or nurse at home?	1	2
Been seen by a mental health worker at home?	1	2

64. Over the last 2 weeks, how often have you been bothered by any of the following problems? **SHOWCARD 27 [if not self completing] AND CODE ONE PER ROW ALL S/C PER ROW**

	Not at all	Several days	More than half the days	Nearly every day
Feeling nervous, anxious or on edge?	1	2	3	4
Not being able to stop or control worrying?	1	2	3	4
Worrying too much about different things?	1	2	3	4
Trouble relaxing?	1	2	3	4
Being so restless that it is hard to sit still?	1	2	3	4
Becoming easily annoyed or irritable?	1	2	3	4
Feeling afraid as if something awful might happen?	1	2	3	4

65. Considering yourself, to what extent do you agree or disagree with the following? **SHOWCARD 28 [if not self completing] AND CODE ONE PER ROW ALL S/C PER ROW**

	Strongly disagree	Disagree	Neither agree or disagree	Agree	Strongly agree
I have been troubled by hearing voices in my head	1	2	3	4	5

66. Considering yourself, to what extent do you agree or disagree with the following? **SHOWCARD 28 [if not self completing] AND CODE ONE PER ROW ALL S/C PER ROW**

	Strongly disagree	Disagree	Neither agree or disagree	Agree	Strongly agree
My friends often tell me to relax and stop worrying about being deceived or harmed.	1	2	3	4	5
I'm often suspicious of other people's intentions towards me.	1	2	3	4	5
People will almost certainly lie to me.	1	2	3	4	5
I believe that some people want to hurt me deliberately.	1	2	3	4	5
You should only trust yourself.	1	2	3	4	5

67. Considering yourself, to what extent do you agree or disagree with the following? **SHOWCARD 28 [if not self completing] AND CODE ONE PER ROW ALL S/C PER ROW**

	Strongly disagree	Disagree	Neither agree or disagree	Agree	Strongly agree
I can easily work out what another person might want to talk about	1	2	3	4	5
I can tell if someone is masking their true emotion	1	2	3	4	5
I can sense if I am intruding, even if the other person does not tell me	1	2	3	4	5
I am good at predicting how someone will feel	1	2	3	4	5
I am good at predicting what someone will do	1	2	3	4	5

68. People think and do many different things when they feel depressed. Please consider each of the following and indicate whether you almost never, sometimes, often or almost always think or do each one when you feel down, sad or depressed. **SHOWCARD 29 [if not self completing] AND CODE ONE PER ROW ALL S/C PER ROW**

	Almost never	Sometimes	Often	Almost always
Think "What am I doing to deserve this?"	1	2	3	4
Think "Why do I always react this way?"	1	2	3	4
Think about a recent situation, wishing it had gone better	1	2	3	4
Think "Why do I have problems other people don't have?"	1	2	3	4
Think "Why can't I handle things better?"	1	2	3	4

NOW HAND THE TABLET BACK TO THE INTERVIEWER.

Section 8: Health and illness

Continuing to think about your own health...

69. To help people say how good or bad their health state is, we have drawn a scale (rather like a thermometer) on which the best state you can imagine is marked 100 and the worst state you can imagine is marked 0. We would like you to indicate on this scale how good or bad your own health is today, in your opinion. (SOURCE: EQ 5D 3L) **ALL LIST**

SHOWCARD 32 AND WRITE NUMBER IN THE BOX:

70. Do you have any physical or mental health conditions or illnesses lasting or expected to last for 12 months or more? **CODE ONE ONLY** (SOURCE: ONS / HSE) **ALL S/C**

Yes	1
No	2
Don't know [spontaneous only]	3
Refused [spontaneous only]	4

71. Now please look at the health conditions listed on this card. Have you ever had any of them over the past 12 months? **SHOWCARD 33 AND CODE ALL THAT APPLY [PROMPT TO ENSURE ALL CONDITIONS ARE MENTIONED AND USE OTHER TO SPECIFY ANY NOT LISTED]** (SOURCE: PSYCHIATRIC MORBIDITY SURVEY) **ALL M/C**

Cancer	1
Diabetes	2
Epilepsy/fits	3
Migraine or other frequent headaches	4
Dementia or Alzheimer's disease	5
Any mental health issue	6
Cataracts / eyesight problems (even if corrected with glasses or contacts)	7
Ear/hearing problems (even if corrected with a hearing aid)	8
Stroke	9
Heart attack/angina	10
High blood pressure	11
Bronchitis/emphysema	12
Asthma	13
Allergies	14
Stomach ulcer or other digestive problems	15
Liver problems	16
Bowel/colon problems	17
Bladder problems/incontinences	18
Arthritis	19
Bone, back joint or muscle problems	20
Gout	21
Skin problems	22
Other, please specify **LIST (SENSE CHECK INTO AN ABOVE CODE)**	95
None of these	96

72. Now we would like you to think about **how you are today**. Choose one option in each group of statements to indicate which statement **best** describes how you are. **SHOWCARD 34 AND CODE ONE FOR EACH** (SOURCE: EQ 5D 3L) **ALL S/C FOR EACH**

Mobility

I have no problems in walking about	1
I have some problems in walking about	2
I am confined to bed	3

Self-care

I have no problems with self-care	1
I have some problems washing or dressing myself	2
I am unable to wash or dress myself	3

Usual activities (e.g. work, study, housework, family or leisure activities)

I have no problems with performing my usual activities	1
I have some problems with performing my usual activities	2
I am unable to perform my usual activities	3

Pain/discomfort

I have no pain or discomfort	1
I have moderate pain or discomfort	2
I have extreme pain or discomfort	3

Anxiety/depression

I am not anxious or depressed	1
I am moderately anxious or depressed	2
I am extremely anxious or depressed	3

73. The questions in this section are about any services you have used over the past 12 months. Please do not consider any occasions you have used these services on behalf of another person. (SOURCE: SANAD2 QoL forms assessing health service utilisation)

a. **Over the past 12 months**, have you done any of the following because of any condition you have or other health reasons? **READ OUT AND CODE ONE PER ROW ALL S/C PER ROW**

b. If yes, please specify how many times in the past 12 months? **WRITE IN NUMBER** Q76a/1 LIST

	Yes	No	If yes, please specify how many times in the past 12 months.
Been to a hospital casualty/A&E department?	1	2	
Been seen by a practice nurse at the GP's surgery?	1	2	
Been seen by the family doctor or another GP at the surgery?	1	2	
Been seen by a nurse at home?	1	2	
Been seen by the family doctor or another GP at home?	1	2	
Been seen by a doctor or nurse at the hospital/clinic outpatient department? [*This will be for a specific appointment*]	1	2	
Been admitted to hospital overnight as an in-patient?	1	2	
Been to a walk-in centre or minor injury unit?	1	2	
Done something different from these? **(please specify)**	1	2	
**ASK Q76c WHERE CODED 1 AT Q76a**			
c. Did you make use of the ambulance service on any of the occasions you have just told us about?	1	2	

74. A. **Over the past 12 months**, have you seen any of the following people through social services or the NHS because of any condition you have or other health reasons? **SHOWCARD 35 AND CODE ALL THAT APPLY** (SOURCE: SANAD2 QoL forms assessing health service utilisation) **ALL M/C**

B. Please specify how many times you have seen them in the past 12 months? **WRITE IN NUMBER Q77a LIST**

		Please specify how many times you have seen them?
A health visitor	1	
A social worker	2	
A physiotherapist	3	
An occupational therapist	4	
A psychological therapist	5	
A counsellor	6	
A dietician	7	
A podiatrist	8	
Some other person (please specify) **LIST (SENSE CHECK INTO AN ABOVE CODE)**	95	
None of these	96	-

75. How many different types of prescribed medication have you taken this week? **WRITE IN NUMBER OR CODE NONE** (SOURCE: HSE) **ALL LIST**

Specify number:	
None	996

ASK Q79-80 WHERE CODED 1+ AT Q78.

76. Have you taken any of these classes of medication in the last week? **SHOWCARD 36 AND CODE ALL THAT APPLY** (SOURCE: HSE) **Q78 1+ M/C**

Cardiovascular medicine	1
Anti-hypertensive medicines	2
Lipid-lowering medicines	3
Antiplatelet medicines	4
Proton pump inhibitors	5
Analgesics and/or NSAIDs	6
Antidepressant medicines	7
Medicines for asthma or COPD	8
Antidiabetic medicines	9
Antibacterial medicines	10
Antipsychotic medicines	11
Contraceptive pill	12
Other (please specify) **LIST**	95
None of these	96
Don’t know	97

77. Do any of your medications cause side effects or bother you in any way? **CODE ONE ONLY** Q78 1+ S/C

Yes	1	Go to Q81
No	2	Go to Q83

78. In what way does it bother you? WRITE IN BELOW Q80/1 LIST (CLEAN COMMENT)

Don't know 97

79. How much does it bothers you? READ OUT AND CODE ONE ONLY Q80/1 S/C

A lot	1
Somewhat	2
A little	3
Never	4

ASK ALL

80. Below is a list of problems that people sometimes have with their medicines. Please indicate how difficult it is, if at all, for you to do each of the following? **SHOWCARD 37 AND CODE ONE PER ROW ALL S/C PER ROW**

	Very difficult	Fairly difficult	Not very difficult	Not difficult at all	Don't know / Not applicable
Open or close the medication's packaging	1	2	3	4	5
Read the print on the packaging	1	2	3	4	5
Remember to take all the pills / dose	1	2	3	4	5
Get your refills in time	1	2	3	4	5
Take more than one medication at the same time	1	2	3	4	5

Section 9: Lifestyle

Finally, thinking about your lifestyle...

81. Which best describes you? If asked, smoking refers to any kind of tobacco, including cigarettes, roll ups, pipe tobacco, cigars, or shisha. **SHOWCARD 38 AND CODE ONE ONLY** (SOURCE: ADAPTED FROM MERSEYSIDE LIFESTYLE SURVEY) **ALL S/C**

I have never smoked	1
I used to smoke occasionally but do not smoke at all now	2
I used to smoke daily but do not smoke at all now	3
I smoke occasionally but not every day	4
I smoke daily	5

82. Do you smoke e-cigarettes? **CODE ONE ONLY ALL S/C**

Yes	1
No	2

83. Do you ever drink alcohol? **CODE ONE ONLY** (SOURCE: MERSEYSIDE LIFESTYLE SURVEY) **ALL S/C**

Yes	1	GO TO Q87
No	2	GO TO Q89

84. On average, how often do you drink alcoholic drinks? **CODE ONE ONLY** (SOURCE: MERSEYSIDE LIFESTYLE SURVEY) **Q86/1 S/C**

Every day of the week	1
Four to six times a week	2
One to three times a week	3
A couple of times a month	4
Less than once a month	5
Don't know/never	6

85. How many of the following alcoholic drinks have you consumed in the past seven days? **SHOWCARD 39 AND WRITE IN NUMBER FOR EACH. WRITE '0' WHERE NOT CONSUMED.** (SOURCE: ADAPTED FROM MERSEYSIDE LIFESTYLE SURVEY) **(Q86/1 LIST FOR EACH)** SCRIPTING INSTRUCTION: SET THIS UP SO THE NUMBER OF UNITS ARE AUTOMATICALLY CALCULATED BASED ON NUMBER OF EACH DRINKS CONSUMED

	WRITE IN NUMBER:	UNITS
Pints of non-alcoholic beer/lager		0
Pints of low alcohol beer/lager		0.75
Pints of normal strength beer/lager		2
Pints of strong beer/lager		2.8
Pints of cider		2
Bottles of alcopops		1.4
Single glasses of spirits (25ml)		1
Standard glasses of wine (175ml)		2
Large glasses of wine (250ml)		3
Single glasses of fortified wines (50ml)		0.9

ASK ALL

86. How much do you weigh without clothes and shoes on? You can tell me in kilograms or in stones and pounds. (SOURCE: HSE)

(WRITE WEIGHT IN STONES/POUNDS OR KILOGRAMS. IF UNSURE, ASK FOR ESTIMATE. IF DON'T KNOW CODE AS 997, IF REFUSED CODE AS 998.) **ALL LIST**

a. Stones / pounds [ ][ ][ ] **Stones** [ ][ ][ ] **Pounds**

Or

b. Kilograms [ ][ ][ ]

87. How tall are you without shoes on? You can tell me in metres and centimetres or in feet and inches. (WRITE IN HEIGHT IN FEET/INCHES OR METRES/CENTIMETRES. IF UNSURE, ASK FOR ESTIMATE. IF DON'T KNOW CODE AS 997, IF REFUSED CODE AS 998.) (SOURCE: HSE) **ALL LIST**

a. Feet / inches [ ][ ][ ] **Feet** [ ][ ][ ] **Inches**

Or

b. Metres / Centimetres [ ][ ][ ] **Metres** [ ][ ][ ] **Centimetres**

ASK Q92 WHERE CODED 2 AT Q1e

88. Please tell us the type and amount of physical activity involved in your work. **SHOWCARD 41 AND CODE ONE ONLY** (SOURCE: GENERAL PRACTICE PHYSICAL ACTIVITY QUESTIONNAIRE) Q1e/2 S/C

I spend most of my time at work sitting (such as in an office)	1
I spend most of my time at work standing or walking. However, my work does not require much intense physical effort (e.g. shop assistant, hairdresser, security guard, childminder, etc.)	2
My work involves definite physical effort including handling of heavy objects and use of tools (e.g. plumber, electrician, carpenter, cleaner, hospital nurse, gardener, postal delivery workers etc.)	3
My work involves vigorous physical activity including handling of very heavy objects (e.g. scaffolder, construction worker, refuse collector, etc.)	4

ASK ALL

89. During the last week, how many hours did you spend on each of the following activities? **CODE ONE ONLY PER ROW** (SOURCE: GENERAL PRACTICE PHYSICAL ACTIVITY QUESTIONNAIRE) **ALL S/C PER ROW**

	None	Some but less than 1 hour	1 hour but less than 3 hours	3 hours or more	Not applicable
Physical exercise such as swimming, jogging, aerobics, football, tennis, gym workout, etc.	1	2	3	4	-
Cycling (including cycling to work) and during leisure time.	1	2	3	4	-
Walking (including walking to work), shopping, etc.	1	2	3	4	-
Housework.	1	2	3	4	-
Childcare.	1	2	3	4	5
Gardening or DIY.	1	2	3	4	-

90. Please indicate how true or untrue the following statement is of you: 'I have high self-esteem'? **SHOWCARD 42 AND CODE ONE ONLY** (SOURCE: UNKNOWN) **ALL S/C**

Not very true of me						Very true of me
1	2	3	4	5	6	7

91. In the future, we would like to contact particular groups of people who have completed the survey (e.g people with particular health conditions or living in particular circumstances) to carry out additional research to improve health or health services. Are you happy for your name and contact details to be kept for this purpose. CODE ONE ONLY **ALL S/C**

Yes, permission given	1
No, permission not given	2

92. **INTERVIEWER TO COMPLETE**: Was the interview conducted in another language (other than English)? **CODE ONE ONLY ALL S/C**

No	1
Yes (specify language)………**LIST**………………..	2

As part of BMG Research's quality control process, my employer will wish to contact some of the people I have interviewed. This is to confirm that I have undertaken the interview in an appropriate manner, and according to market research practice. Are you happy to provide me with your name, confirm your address and provide me with a contact telephone number. This information will not be passed on, or used for any purpose other than our quality control processes unless you provided permission. Your details will be deleted as soon as our quality controls process ends.

**READ :** Thank you, those are all the questions I have.<br>If you are concerned about whether BMG is a genuine market research agency you can call the Market Research Society on 0500 396 999 during office hours. Finally I need you to verify that you have taken part in this survey and that I have accurately recorded your comments, by signing the following statement:

IMPORTANT: TAKE CARE TO RECORD RESPONDENT NAME AND ADDRESS DETAILS ACCURATELY.

RESPONDENT'S NAME:	
ADDRESS: (Address Line 1)	
(Address Line 2)	
(Address Line 3)	
(Postal Town)	
(County)	
POSTCODE: (**ESSENTIAL**)	
TELEPHONE: (INCLUDING **STD**) (**ESSENTIAL**)	

REMEMBER TO HAND OUT THANK YOU LEAFLET AND SUPPORT LEAFLET
